# The Role of Male Consent in Assisted Reproductive Technology Procedures: an Examination of Japanese Court Cases

**DOI:** 10.1007/s41649-023-00274-1

**Published:** 2024-01-18

**Authors:** Yuko Muraoka, Minori Kokado, Kazuto Kato

**Affiliations:** 1 Department of Biomedical Ethics and Public Policy, Graduate School of Medicine, Osaka University, Osaka, Japan; 2 Graduate School of Humanities, Osaka University, Osaka, Japan

**Keywords:** Assisted reproductive technology, Cryopreservation, Male consent, Reproductive rights, Right of self-determination, Postmortem conception

## Abstract

With the development of assisted reproductive technologies, medical, ethical, legal, and social issues have arisen that did not exist when natural conception was the only means of childbirth. In Japan, men tend to believe that assisted reproductive technologies are not directly related to them, with the literature showing that men are often reluctant to be involved in fertility treatment processes. To better understand this situation, this study analyzes the role of male consent during assisted reproductive technology procedures in Japan. First, we examined Japanese court cases that dealt with issues related to male consent during assisted reproductive technology procedures and identified three situations in which problems related to male consent during such procedures may arise. Next, we analyzed the background of such issues and the implications of the lack of consent regarding men’s reproductive rights. Finally, we explored the need for legislation on assisted reproductive technologies. The study concludes that discussions on the scope of male partner rights in assisted reproductive technology procedures are key for minimizing unnecessary conflict between partners, thus ensuring both the rights of women who wish to have children and the welfare of their children.

**Supplementary Information:**

The online version contains supplementary material available at 10.1007/s41649-023-00274-1.

## Introduction

Since the birth of the first baby through in vitro fertilization (IVF) in the UK in 1978, the use of assisted reproductive technologies (ART) has spread rapidly to many parts of the world. In response to this development, countries have implemented various forms of regulation related to ART (Council of Europe 2012; Ministry of Justice Japan 2021). In Germany, for example, there is no single code that comprehensively regulates ART, and the relevant provisions are scattered across several laws and other norms. However, there are guidelines^1^ that provide comprehensive and specific provisions on gamete harvesting and transplantation. In France, the law^2^ also clearly states what ART are available, further specifying that written consent is required for the procedure. These examples demonstrate that ART regulation methods and content vary across nations. In addition, the use of ART has given rise to numerous medical, ethical, legal, and social issues that did not exist when natural pregnancy was the only means of childbirth (Aono 2004; Soini et al. 2006).

Women are overwhelmingly the focus of research in the field of infertility and reproduction. Studies focusing on male consent are scarce, despite the fact that some cases related to the handling of sperm and frozen embryos have been brought to the European Court of Human Rights (Culley et al. 2013; European Court of Human Rights 2007; Ishikawa and Okazaki 2014). This imbalance can also be seen in the context of Japan. Japan ranks second only to China in terms of the number of ART cycles performed (Adamson et al. 2022). However, Japanese men do not often see the ART issue to be directly related to them; for example, in one study of men receiving treatment for infertility, all participants indicated that they were receiving treatment for their wives (Takeya 2021a, b). Furthermore, there is a scarcity of research that wholistically analyzes the role of male consent in ART procedures and the problems that arise when this consent is lacking.

To contribute to the broader understanding of this issue, this study examines the role of male consent in ART in Japan and proposes measures to prevent unnecessary conflict. More specifically, we analyze Japanese court cases concerned with male consent issues in ART procedures and identify situations in which such issues may occur. Thereafter, we explore the background of such issues and the implications for men’s reproductive rights when there is a lack of consent in ART procedures.

It should be noted that all the Japanese court cases analyzed in this study were cases in which biologically male–female couples used ART. As such, this study examines issues related to the use of ART within the context of biologically male–female couples and does not consider cases involving non-heterosexual couples and transgender/non-binary individuals. While it was not within the scope of this study, we acknowledge that there is a significant need to promote discourse and understanding surrounding such diversities, especially considering the reality that the reproductive rights of non-heterosexual couples and transgender/non-binary individuals are severely limited in many societies.

## Background: Current Status of ART in Japan

ART systems and policies differ from country to country. To explain the context of the Japanese court cases examined in this study, we review the current status of the ART system in Japan.

Japan consists of 47 prefectures and has a population of 125.28 million (Government of Japan 2022). Medical services are based on a universal health insurance system (Ministry of Health, Labour and Welfare Japan [MHLW] 2012), where patients pay a portion of their medical costs (in most cases, the co-pay is 30%), and the rest is covered by insurance. Individuals seeking medical care are free to research and choose their own healthcare providers.

For many years, ART was, in principle, not covered by Japan’s universal health insurance system but was rather self-funded; therefore, patients bore the full cost of treatment.^3^ In a survey of those who had undergone IVF or intracytoplasmic sperm injection, most respondents spent more than JPY 1 million on medical expenses, and nearly 30% spent more than JPY 2 million (MHLW 2021a).^4^ However, since April 2022, ART has been covered by universal health insurance, albeit with a few restrictions on age (treatment must begin by age 43 for women) and frequency (up to a total of six treatments for those under the age of 40 and up to three treatments for those between 40 and 43 for women; MHLW 2022). No current restrictions on men’s age exist.

The number of couples undergoing fertility treatment in Japan has been increasing. In 2018, 56,979 (approximately 1 in 16) or 6.2% of the total 918,400 births occurred via ART (MHLW 2021b; JSOG 2018). Furthermore, a 2015 survey found that one in 5.5 couples had undergone fertility testing or treatment (National Institute of Population and Social Security Research 2015). The recipients of ART are usually “couples who strongly desire to have a child,” including couples in de facto marriages (JSOG 2022).

While the need for ART laws has been discussed in Japan since the 1990s, such laws have not yet been implemented. In 1998, the Ministry of Health and Welfare (now the MHLW) established an “Expert Committee on Assisted Reproductive Technology.” In 2000, a report compiled by the committee called for ART legislation within the following 3 years (MHLW 2000). However, owing to political opposition (Minami 2011), the report was not submitted to the Diet and was subsequently dropped. Almost 20 years later, on December 4, 2020, a law regarding the parent–child relationship of a child born through third-party gamete donation was enacted (Act No.76 of 2020).^5^ However, this act did not include information on ART using a gamete created between husband and wife or between partners (Ministry of Justice Japan 2020).

As no other laws currently exist, ART is conducted exclusively according to the guidelines of professional associations. The Japan Society of Obstetrics and Gynecology (JSOG) guidelines state that the consent of all parties must be obtained for each ART procedure (JSOG 2014). The Japanese Society for Reproductive Medicine also requires the consent of both spouses at the time of embryo transfer (Japanese Society for Reproductive Medicine 2022). This is a similar policy to that of several countries that Japan surveyed when enacting Act No.76 of 2020, including France, the UK, and Austria.

## Survey of Court Cases

### Survey and Organization of Japanese Court Cases

As aforementioned, Japanese guidelines require the consent of the male partner during ART procedures, including embryo transfer. To examine this process, we reviewed court cases that dealt with issues related to male consent through the Japanese case law databases Westlaw Japan and D-1 Law. Newspaper reports were also included in this review. The court cases studied took place from April 1891 to May 2022, which is the entire period covered by these databases. However, all court cases in which male consent was an issue occurred from 2000 onward. The keywords used for the database search were “consent” and one of the following combinations: “ART,” “fertility treatment,” “in vitro fertilization,” or “fertilized egg/fertilized embryo.”

**Fig. 1:**
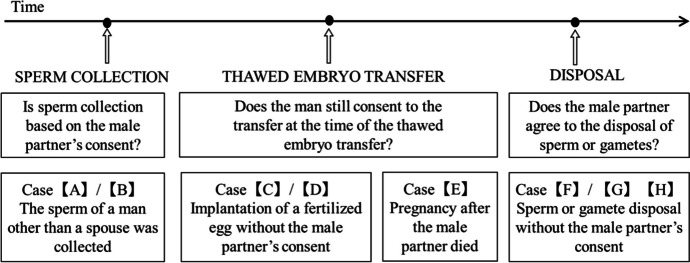
Time points for the lawsuit cases in which male consent was an issue

The review revealed eight court cases in which disputes were related to male consent during ART procedures (cases A through H; see the Supplementary Information file). The eight court cases were categorized based on the point at which consent by the male partner became an issue during ART procedures. As shown in Fig. 1, situations in which male consent was an issue were categorized into three time points: (1) at the time of sperm collection, (2) at the time of frozen embryo transfer (including cases of postmortem conception), and (3) at the time of sperm or frozen embryo disposal.

### Category 1: Examples of Problematic Consent at the Time of Sperm Collection

#### Case A

In 2010, a married woman intentionally provided sperm derived from a man who was not her husband to a medical institution as her husband’s sperm for the purpose of artificial insemination. No direct identification/confirmation of the husband was performed by the medical institution when handling the sperm, and the implantation of embryos created with the sperm was carried out by the medical institution in 2011. The husband sued the medical institution, claiming that it had “an obligation to confirm that the sperm used for the ART belonged to the right person at every step of the ART process; namely when the sperm is collected, received, or used.” Damages against the medical institution were then claimed based on default, with allegations that they had breached their obligations. At the same time, the husband also contested seeking disclosure of information and other matters concerning the use of the sperm. The first court hearing dismissed the husband’s claims on the ground that he had failed to prove that the sperm brought to the medical institution was not the husband’s sperm (Tokyo District Court 2016). The appeals court found that the sperm in question was the sperm of a person other than the husband. In that case, however, it concluded that the husband was not a party to the medical treatment contract with the medical institution and that the medical institution had no obligation to the husband. As a result, the husband’s claims were remained dismissed (Tokyo High Court 2017).

#### Case B

In 2012, a single woman intentionally provided the sperm of a married man to a medical institution as her husband’s sperm, and a child was born through artificial insemination (Tokyo District Court 2012). The wife of the man whose sperm was used sued the woman for committing a tort, claiming that the artificial insemination in question was an illegal act that violated peaceful family life (Civil Cord Article 709, 715). The man’s wife also claimed that the medical institution was jointly liable with the single woman. The court approved the man’s wife’s claim against the woman and ordered her to pay JPY 2 million as compensation. However, the court denied that the medical institution was responsible.^6^ In this case, the man had consented to the sperm collection; therefore, strictly speaking, this is not a case where male consent was an issue. However, this case is similar to case A in that the woman brought the sperm alone to the medical institution, and no direct confirmation or acquisition of consent from the man was conducted. In addition, this case involved artificial insemination between a married man and a woman other than his wife, which was viewed an act of infidelity, a situation not accounted for by the ART guidelines in Japan.

### Category 2: Examples of Problematic Consent at the Time of Thawed Embryo Transfer

There were two cases in which frozen embryos created between a couple during previous fertility treatment were later transferred to the wife without the husband’s permission, resulting in the birth of a child (cases C and D). Two different disputes arose from each of these cases: a family case, in which the husband claimed no legal parent–child relationship to the child, and a civil case, where the husband claimed damages against the wife and the medical institution, claiming that his “rights” had been violated.

#### Case C

The family case [C] was reportedly the first case in Japan in which unauthorized fertilized embryo transfer and the legal parent–child relationship became an issue (Mainichi Shimbun 2017b). The couple underwent IVF and gave birth to their first son in October 2010. The remaining fertilized eggs were cryopreserved. The couple then separated, and in 2014, the wife wanted to have a second child, but the husband refused. In May of the same year, the wife forged her husband’s signature on a consent form, attempted to transfer the frozen fertilized eggs, and became pregnant in July of the same year. The husband denied a legal parent–child relationship with the child and claimed, “Rebutting the Presumption of Child in Wedlock” (Civil Code Article 774). The Nara District Court, which was the court of first instance, rejected the husband’s claim and recognized the legal parent–child relationship between the husband and the child on the grounds that the 1-year statute of limitations (Civil Code Article 777) had already passed at the time the husband filed his suit. However, regarding ART, the court indicated for the first time the standard that “the husband’s consent is required at the time of transfer of fertilized eggs” (Inaba 2021).The Osaka High Court of Appeals reached the same conclusion, rejecting the husband’s claim and recognizing the legal parent–child relationship (Sankei Shimbun 2018). However, the necessity of the husband’s consent at the time of fertilized egg transfer was not mentioned.

In the civil case [C], the husband filed a tort claim against his wife and the clinic for damages (Civil Cord Article 709, 715), but the case was terminated by settlement and the outcome was not made public (Mainichi Shimbun 2017a).

#### Case D

Similarly, case D was a case in which frozen embryos were implanted in a woman without her husband’s consent. In the family court case [D] (Osaka Family Court 2019), the husband argued that there was no legal parent–child relationship between him and the child by asserting both a claim of “Rebutting the Presumption of Child in Wedlock” (Civil Code Article 774) and a claim of action seeking a declaratory judgment as to whether a biological parent and child relationship exists (Personal Status Litigation Act (Act No.109 of 2003) Article 2 (ii)). In this case, the issue of “whether the husband’s consent at the time of transplantation is a requirement in recognizing a legal parent–child relationship” was contested as an important issue. The court held that “in the absence of legislation, it is reasonable to interpret the legal parent–child relationship in the same way as for children born through natural reproduction.” In other words, the court concluded that “male consent at the time of embryo transfer” is not necessarily required when considering the legal parent–child relationship with a child born through ART. As a result, the court recognized the legal parent–child relationship.

In the civil case [D] (Osaka District Court 2020), the husband sued his wife and the medical institution based on tortious conduct (Civil Cord Article 709, 715). The husband won the case, and the wife and medical institution were obligated to pay JPY 8 million as compensation for the husband’s emotional distress, which is a high level of compensation in Japan (the amount was later reduced to JPY 5 million at the appeals court; Osaka High Court 2020). The Osaka District Court, the court of first instance, concluded that “it is clear from the nature of the matter that the plaintiff’s [husband’s] consent was required for this transplantation” and that “the defendant [wife] is liable to the plaintiff for tortious acts, including violating his right to self-determination as to whether to have the child in question with the defendant.”

#### Case E

Postmortem conception can also be considered an instance in which the husband’s consent at the time of thawed embryo transfer is an issue. In case E, the issue was the legal parent–child relationship regarding a child born by artificial insemination after the husband’s death. The wife filed an action for recognition (Civil Code Article 787), naming the prosecutor as a defendant.^7^ The appeals court stated that “in order for a claim for recognition of a child born through artificial insemination to be recognized, it is necessary and sufficient that, in addition to the existence of a natural consanguinity between the child and the de facto father, the de facto father’s consent to such conception be satisfied.” The court affirmed the parent–child relationship, with an emphasis on the consent of the male partner (Takamatsu High Court 2004). However, the appeal court’s conclusion was reversed by the Supreme Court (Supreme Court 2006), which stated, “In the absence of legislation, the formation of a legal parent–child relationship between a posthumously conceived child and the deceased father cannot be recognized.”

### Category 3: Examples of Problematic Consent at the Time of Disposal

#### Case F

Consent is also an issue in situations where frozen sperm and fertilized eggs are to be disposed of. In case F, a medical institution disposed of sperm at the wife’s request, and the issue was whether the husband, the sperm donor, gave his consent (Tokyo District Court 2017). The husband claimed that he suffered emotional distress when his sperm was disposed of without his permission and demanded compensation from the medical institution (Civil Code Article 709, 715). The court recognized that the wife had prepared the “Application for Disposal of Cryopreserved Sperm,” which should have been prepared by the husband. However, the court also determined that the husband’s agreement to the wife’s preparation of this consent form was inferred, and the husband lost the case.

#### Case G and Case H

There were also some reported cases (cases G and H) in which a medical institution mistakenly disposed of fertilized eggs without confirming that both spouses consented to the disposal (Mainichi Shimbun 2019b). In case G, the couple froze six fertilized eggs in 2007, but three of them were mistakenly discarded by the clinic in 2008. The couple filed a claim for compensation with the clinic based on emotional distress (Civil Code Article 709, 715). According to the newspaper report (Asahi Shimbun Digital 2020), the medical institution apologized and paid the couple a settlement.

Case H is similarly a case in which the clinic lost one of the frozen embryos and the couple claimed emotional compensation from the clinic (Mainichi Shimbun 2019a). The case ended in settlement, and details were not available.

## Discussion

The majority of court cases examined in this study comprised situations in which the man and woman were in conflict over the use or disposal of a gamete. The exceptions included case E, which related to postmortem conception, and cases G and H, which involved procedural errors by a medical institution. Moreover, cases C and D, which resulted in the birth of a child, raise important questions regarding the recognition of a legal parent–child relationship between the male partner who did not consent to the embryo transfer and the child that was born.

These results suggest that carefully considering the role of the male partner’s consent is necessary to minimize conflict in ART procedures. We believe that minimizing such conflict is important for protecting the rights of both women and men, as well as for avoiding complications related to the legal status of the unborn child. We discuss these issues below, along with the contextual background that contributes to the lack of male consent.

### Implications for the Reproductive Rights of the Male Partner as a Result of Lack of Consent

As aforementioned, Japanese guidelines on ART stipulate that consent by the male partner is required for every thawed embryo transfer. However, our results show that there are cases where this consent is not always obtained. Furthermore, Japanese courts do not require the consent of the male partner when determining the legal parent–child relationship of a child born through ART. Thus, the male partner would be obligated as a legal parent if a child is born using his gametes, even if he is not at all involved in the thawed embryo transfer. Does this not constitute an infringement on the reproductive rights of the male partner? This proposition requires a clarification of what exactly men’s reproductive rights are.

As explained by the Programme of Action from the International Conference on Population Development (1999), reproductive rights “recognize the right of all couples and individuals to be free and responsible for determining the number, spacing, and timing of their children, and to have the information and means to do so, as well as the right to the highest possible standard of sexual and reproductive health”. The right to decide with whom to have a child can be considered an aspect of reproductive rights. While this applies to both men and women, the focus of reproductive rights is often centered on women (Culley et al. 2013), particularly in terms of women’s fundamental human rights; it is often questioned whether the right to “bear a child or not” is a direct reproductive right of men (Tsuge 2000; Tsujimura 2021). This is for several reasons, including the notion that women^8^ are often the ones who bear a greater physical burden (e.g., pregnancy and childbirth), as well as the persistence of widespread inequality between men and women in many parts of society. In particular, intense debate on the right to abortion has further highlighted the clear need to protect women’s reproductive rights.

However, while abortion involves procedures that directly affect the woman’s body, the male partner’s refusal to consent to the transfer of embryos does not. Furthermore, the tendency to view reproduction, contraception, and childbirth as inextricably linked with women leads not only to an unequal burden of reproductive responsibility, but also to the marginalization of the role of the male partner in parenthood and the violation of the reproductive right to decide whether to have children (Annandale and Clark 1996). In other words, efforts to protect women’s reproductive rights should not come at the cost of overlooking men’s reproductive rights. Furthermore, the inclusion of discourse on men’s reproductive rights is an important aspect of protecting the rights of both women and men, as well as avoiding complications regarding the legal status of the unborn child.

The decisions of the court cases analyzed in this study indicate that transferring embryos without consent violates the male partner’s right to self-determination. In civil case [D], the court held that the man’s “right to self-determination as to whether or not to have a child with the said woman [ex-wife]” existed and was violated. It was stated that “because bearing and raising children is a fundamental part of the right to personal survival for those who wish to do so, the right to decide whether or not to bear and raise children should be respected as a right that constitutes a component of personal rights in light of the legal intent of Article 13 of the Constitution, which guarantees the right to the pursuit of happiness.” Several other court decisions have also held that “whether or not to bear and raise children” falls within personal rights, regardless of gender (Sendai District Court 2019). This can be interpreted to include the “right to not bear and raise children,” that is, the right of a male partner to “not desire a pregnancy (by means of a fused embryo transfer)” with the woman in question, as a subject of legal protection.

The concept of the male partner’s right to choose whether to have a child is not unique to Japan. In the USA, there is active debate about whether the rights of an individual that does not wish to become a genetic parent of a child born from frozen embryos should be recognized (Cohen 2008). The European Court of Human Rights has also dealt with a case in which the man sought to dispose of embryos after the end of the couple’s relationship and the woman sought an injunction against it (European Court of Human Rights 2007). In this case, the woman had her ovaries removed after the frozen embryos were created, meaning that if the frozen embryos were discarded, the woman in question would lose any means to have biological children. However, while noting the woman’s plight, the court stated that the rights both “to be a parent” and “not to be a parent” must be respected and allowed the man to request the disposal of the frozen embryos. This statement shows consideration of the reproductive rights of the male partner “not to be a parent”: What is protected here can be understood to be the right of the male partner to self-determination.

Furthermore, a case resulting in the birth of a child and recognition of the legal parent–child relationship without consent can be considered a violation of the right to self-determination, not only as a personal right, but also as a property right (Osaka Family Court 2019). Some countries have planned for such situations and have stipulated laws in advance. For example, regarding the use of frozen embryos after divorce, the Uniform Parentage Act of 2000 in the USA specifies “that the former spouse shall not be the parent in the event of divorce prior to conception, unless otherwise expressly agreed.” This law states that a man has neither rights nor obligations as a legal father. However, in countries and regions where there is no such legal provision, if the male partner is recognized as the legal father of the child, he bears the financial burden of child support and other support obligations. The child would also be entitled to inheritance rights. Both issues can have a significant influence on a man’s life for a long period after the child is born. While there are numerous situations in which the reproductive rights of women have not yet been secured, failing to protect the reproductive rights of men also has significant consequences.

### Significance of Consent: Does Consent for Frozen Embryo Creation not Include Consent for Embryo Transfer?

There is a suggestion that additional consent at the time of embryo transfer is not necessary in ART procedures. It has also been suggested that consent for the birth of the child is implied at the time of the initial consent for embryo creation (Inaba 2021). However, there are two main reasons for requiring additional consent at the time of transferring frozen embryos.

First, ART procedures are a “medical intervention,” and frozen embryo creation is a medical treatment for both the female and the male partner. Article 6 of the Universal Declaration of Human Rights on Bioethics and Human Rights states that “any preventive, diagnostic, and therapeutic medical intervention is only to be carried out with the prior, free, and informed consent of the person concerned, based on adequate information” (UNESCO 2005). The consent should, where appropriate, be expressed and may be withdrawn by the person concerned at any time and for any reason without disadvantage or prejudice. Consent for medical intervention must be free, explicit, and revocable. Furthermore, considering the time gap between the separation of the gametes from the body and embryo transfer, it follows that individual consent is required even at the time of embryo transfer.

Second, the consent obtained at the time of frozen embryo creation differs from the consent obtained at the time of embryo transfer. In other words, the male partner’s consent at the time of frozen embryo creation can be considered as consent to the medical treatment of separating gametes from the body and creating a frozen fertilized embryo. Certainly, frozen embryo creation is performed with the initial intention of having a child; however, there is no possibility of pregnancy or birth if the embryo remains frozen (Purvis 2021). In contrast, embryo transfer is a specific medical treatment that leads to pregnancy and childbirth. The consent by the male partner at this point can be defined as the desire or willingness to allow the gamete to be implanted into the woman’s uterus, resulting in pregnancy and delivery. This is different from the purpose of obtaining consent for frozen embryo creation.

Examining examples from other countries reveals that in some cases, such as Italy (Italian Constitutional Court 2023),^9^ the withdrawal of consent by the male after fertilization is restricted. In other cases, there are disputes over interpretations of the time length that consent can be withdrawn. For example, in Switzerland the consent of both partners is required for reactivation of cryopreserved fertilized eggs, but it has been interpreted that withdrawal of consent by the male partner is not possible after reactivation of the cryopreserved fertilized eggs for transfer (Ministry of Justice Japan 2021). However, the two points discussed above suggest that the male partner should be allowed to withdraw his consent for the use of embryos after their creation.

### Background to the Male Partner’s Absence from Medical Practice

To protect the right of the male partner to self-determination regarding “whether or not to become a parent,” their consent must be reaffirmed at the time of “embryo transfer.” This requirement is clearly stated by Japanese guidelines. However, as shown in some of the cases reviewed in this study, consent from the male partner is not always confirmed at the time of transplantation (cases C and D). The common factor in all of these cases is that the male partner was physically absent when the treatment was performed in medical institutions. Such treatments include sperm collection by the institution (cases A and B) and embryo disposal (cases G and H). For example, when one woman received a thawed embryo transfer in the hospital, her male partner was not present. In another case, it was the woman who brought her male partner’s sperm to the hospital and prepared the documentation for sperm disposal. These cases suggest that Japanese men have the tendency to think of ART as an issue not directly related to them.

Despite the lack of male partners’ presence at the medical institution, ART treatments continue. This is remarkable considering that under normal circumstances, it is impossible for a procedure to proceed without a patient being present at the medical institution. Why, then, does this occur only with ART? One reason could be that ART treatment, which is performed to achieve pregnancy, primarily affects women: The average duration of medical facility visits required for ART procedures is 6–12 days for women and only 0–1 day for men (MHLW 2018).

Another factor is medical institutions’ approach to infertility treatment. Previous studies have suggested that medical institutions in Japan tend to perceive infertility as the woman’s problem (Abe and Tomita 2002), and information that should be explained directly to men is sometimes handled by the woman. A Japanese survey reported that in situations where sperm test results are received at an obstetrics and gynecology medical institution, the husband is not present in about half of the cases; instead, the wife receives an explanation from the doctor alone (MHLW 2016). In any other type of medical practice, it would be highly unusual for a stranger (the wife) to be the recipient of an explanation about another patient’s (the husband’s) medical treatment. Even in male infertility treatment, doctors tend to focus more attention on the female rather than the male partner, with the female partner usually more actively involved in the treatment (Takeya 2021b). Based on the perception that infertility treatment is for women, medical institutions themselves give priority to the female partner.

This perception is rooted in deeper societal gender norms, especially in Japan where the work environment perpetuates a traditional gender role division. According to a study published by the OECD in 2020, the gap between paid work time for men versus women in Japan is 1.7 h, which is the highest gap of all countries surveyed. Gender norms and traditional understandings of the division of labor can contribute to the perception that there is no need for men to be involved in ART procedures.

### The Male Partner’s Will and Initiative

In addition to the aforementioned factors, the problem of the lack of male consent can also be linked to a lack of awareness amongst men regarding their agency in ART procedures. Japanese surveys have shown that some male parties do not consider fertility treatment to be a problem directly related to them (Takeya 2021a, b). Similarly, cases C and D can also be considered an example of the male partners’ failure to express their intentions to be involved. If the male partners in these cases had requested that the medical institutions prohibit the use of the frozen embryos when the couples’ relationship came to an end, the medical institutions would not have been able to perform the embryo transfer, and the children would not have been born against the will of the male partners. In these cases, the male parties failed to express their desires, despite the fact that they had the “privilege [unlike in the instance of natural pregnancy] of being able to stop ART without harming the woman’s body before embryo transfer and pregnancy” (Inaba 2022).

What then lies behind the lack of the male party’s proactiveness? One factor is that pregnancy and childbirth are physically related to women. Another possible factor is the lack of discussion on what rights are granted to the male party, the “non-childbearing sex,” in reproductive situations in the first place.

Men, who are indispensable to reproduction, should be granted the right to self-determination in the area of sex and reproduction to the extent that it does not infringe upon women’s rights. The specifics of this right to self-determination have not been adequately discussed. However, with the recent development of ART, there are now options for pregnancy and childbirth that did not previously exist, and men are increasingly making reproductive decisions without infringing on women’s rights. For example, in the case of natural conception, it is unacceptable for a man to ask a woman to terminate her pregnancy once she becomes pregnant as this would force her to undergo an abortion procedure, which is a surgical invasion of the body. However, as abovementioned, in ART, there is an opportunity to stop the procedure by way of non-consent to transplantation without directly violating the woman’s body. Other examples of situations in which the man’s will is an issue include whether a couple wishing to have a baby should undergo treatment when the man is the cause of infertility or whether they should choose artificial insemination with donor sperm when artificial insemination with their male partner’s sperm is not an option. Furthermore, what is the position of the male partner in preimplantation diagnosis for selecting embryos that are free of genetic diseases? Moreover, how is a man involved in whether to undergo prenatal diagnosis for the fetus? These considerations are all directly or indirectly connected to a male party having or not having a child and are considered to be important issues concerning the personal interests of the male party concerned.

## Implications: Ensuring the Male Party’s Involvement in ART

Based on the above analysis, we now discuss possible ways to strengthen the involvement of the male partner in ART procedures. It should be noted that the discussion of male partners’ rights in pregnancy and childbirth is simultaneously a discussion of their obligations as fathers or husbands. In this regard, such a discussion does not imply the restriction of women’s rights, but rather is a necessary component of protecting the rights of women who become mothers and ensuring the welfare of the unborn child.

### Rules for Securing Consent at the Time of Embryo Transfer

In defining the handling of male consent in situations involving the birth of a new life, it is not sufficient to use guidelines that are not enforceable by law. In Japan, there has been a public desire for the legal regulation of ART for more than 20 years, which has resulted in Act No.76 of 2020. However, this law does not include specific provisions regarding how consent should be obtained. As seen in this study’s review, there have been cases in which the biological father denied the parent–child relationship as a result of his consent not being properly obtained. Furthermore, rules should be established that dictate how medical institutions should confirm the male partner’s consent, including the use of interviews, telephone calls, and other means of discussion. From the perspective of the unborn child’s welfare, awareness of the importance of male consent and taking steps to ensure that it is obtained at the necessary points of the process are required.

### Bringing Attention to the Male Partner’s Right to Self-Determination for Having a Child

In much of legal history and constitutional law, globally, reproductive rights have become synonymous with women (Ziegler 2020). Discussions on the reproductive rights of men are noticeably insufficient in constitutional law (Inaba 2022). This is not a situation limited to a few countries but is the general trend worldwide. Further, the discussion of reproductive rights in the context of women is not limited to the fields of legal history and constitutional law (Tsuge 2012). According to Culley et al. (2013), further research on the male partner is needed in all areas, including perceptions of infertility and infertility-treatment-seeking behaviors, experiences of treatment, information and support needs, decisions to end treatment, fatherhood, the post-assisted conception period, and the motivations and experiences of sperm donors and men who seek fatherhood through surrogacy or co-parenting. Rather than viewing ART and infertility as issues unique to women, discussing the roles and obligations of men could lead to achieving gender equality and protecting the welfare of the unborn child.

With current ART developments, we believe that it is necessary to reconstruct the male partner’s reproductive rights, especially considering that the means of having children are no longer limited to natural conception. In line with the notion of women’s reproductive rights that women should not be treated as “tools of childbearing,” transplanting frozen embryos from men who are unwilling to have children can be thought of in the same way (Kodama 2006). This new, problematic issue arises because of medical developments that have allowed gametes to exist outside the physical body.

At the same time, there is an argument that the agency of the male partner does not necessarily need to be equal to that of the female partner. This is because there is still an imbalance in the physical burden between the sexes in ART: The physical burden on the woman in the egg retrieval situation is greater than that on the man in the sperm retrieval situation. Even in the case of male infertility, most assisted reproduction medical processes directly involve women and are invasive to the female body (Tsuge 2012). Furthermore, it may also be necessary to consider the imbalance resulting from the fact that the female partner’s reproductive period is more limited than that of the male partner. Given this reproductive asymmetry and imbalance, where the woman is required to bear the (physical) burden unilaterally, it would be unfair for the man to have an equal say. Consequently, some posit that the “right to fulfill his duty [to protect the rights of women and children]” is a man’s reproductive right, which is a type of social right (Numazaki 2000). There is room to focus on the individual and construct the content of the male partner’s right to self-determination as “the right to not have a child with a partner.” This is precisely where civil case [D] stated that the husband had the “right of self-determination as to whether or not to have the child in question with the wife.” Careful discussion is needed regarding how these circumstances should be dealt with when considering the rights of male partners.

## Limitations

It should be noted that all the Japanese court cases analyzed in this study were cases in which biologically male–female couples used ART. As such, this study examined issues related to the use of ART within the context of biologically male–female couples and did not consider cases involving non-heterosexual couples and transgender/non-binary individuals. While it was not within the scope of this study, we acknowledge that there is a significant need to promote discourse and understanding surrounding such diversities, especially considering the reality that the reproductive rights of non-heterosexual couples and transgender/non-binary individuals are severely limited in many societies.

## Conclusion

This study investigated the current situation of the lack of male consent in ART in Japan, a country with a significantly high number of fertility treatments. The court cases analyzed in this study seem to be just the tip of the iceberg. In addition to inadequate laws and regulations, we highlight that the stereotypical concept of the division of labor roles in which men work and women bear and raise children may still exist in society, especially in Japan. We also note a tendency toward a clear division of labor in Japanese work environments, especially in terms of men’s and women’s roles. Our examination elucidates the need for further discussion on the reproductive rights of men and the lack thereof, which indirectly contributes to the lack of male ownership over childbirth.

With the development and spread of ART, the relationship between the rights of the male and female partners in reproductive settings has become increasingly complex. It is important to discuss the range of the male partner’s rights to self-determination in the context of ART to protect both the rights of female partners who wish to have children and the welfare of their unborn children.

### Supplementary Information

Below is the link to the electronic supplementary material.

Supplementary file1 (XLSX 22 KB)

## Data Availability

Data sharing is not applicable to this article as no datasets were generated or analyzed during the current study.

## Footnotes

1 Richtlinie zur Entnahme und Übertragung von menschlichen Keimzellen im Rahmen der assistierten Reproduktion, Deutsches Ärzteblatt 2018, A 1.

2 Loi n° 2021–1017 du 2 août 2021 relative à la bioéthique. https://www.legifrance.gouv.fr/jorf/id/JORFTEXT000043884384/. Accessed 9 November 2023.

3 A local government subsidy program subsidized a portion of the cost of infertility treatment. However, the requirements for receiving the subsidy were strict, and the system was not open to everyone undergoing infertility treatment.

4 As of 6 November 2023, the exchange rate between the JPY and the US dollar and the Euro is approximately JPY 150 for 1 US dollar and JPY 160 for 1 Euro. Therefore, JPY 1 million is equivalent to approximately 6686 USD and 6234 EUR, and JPY 2 million is equivalent to approximately 13,372 USD and 12,468 EUR.

5 Act on Assisted Reproductive Technology Offering and the Special Provisions of the Civil Code Related to the Parent–Child Relationship of a Child Born As a Result of the Treatment (Act No.76 of 2020).

6 In this trial, the wife’s argument was that the woman and the medical institution jointly violated her rights and were jointly and severally liable for it, but only the woman was actually a party to the trial as a defendant.

7 In Japanese lawsuits for recognition, it is stipulated that the public prosecutor is to be named as the defendant if the person that should be named as a defendant is dead (Personal Status Litigation Act Article 12 (3)).

8 A person of any gender can produce eggs and/or have a uterus, and this article uses inclusive language wherever possible. Here, we mean to imply a gendered treatment of potential parents by clinics; thus, we use the term “women” deliberately.

9 The Italian Constitutional Court has ruled that women will be able to use embryos conceived with their husband or partner, even if they do not have their consent or are divorced.
